# *THBS1*-Mediated Degradation of Collagen via the PI3K/AKT Pathway Facilitates the Metastasis and Poor Prognosis of OSCC

**DOI:** 10.3390/ijms241713312

**Published:** 2023-08-28

**Authors:** Zhihao Wen, Yuxiao Zhang, Xiangyao Wang, Yaxin Wu, Jing Mao, Qilin Li, Shiqiang Gong

**Affiliations:** 1 Department of Stomatology, Tongji Hospital, Tongji Medical College, Huazhong University of Science and Technology, Wuhan 430030, China; kqys_wzh@163.com (Z.W.); m202372648@hust.edu.cn (Y.Z.); m202076256@hust.edu.cn (X.W.); yaxinwu@163.com (Y.W.); maojing@hust.edu.cn (J.M.); 2 School of Stomatology, Tongji Medical College, Huazhong University of Science and Technology, Wuhan 430030, China; 3 Hubei Province Key Laboratory of Oral and Maxillofacial Development and Regeneration, Wuhan 430022, China

**Keywords:** oral squamous cell carcinoma (OSCC), extracellular matrix (ECM), collagen degradation, prognosis, immunotherapy

## Abstract

Oral squamous cell carcinoma (OSCC) is a prevalent form of malignant tumor, characterized by a persistently high incidence and mortality rate. The extracellular matrix (ECM) plays a crucial role in the initiation, progression, and diverse biological behaviors of OSCC, facilitated by mechanisms such as providing structural support, promoting cell migration and invasion, regulating cell morphology, and modulating signal transduction. This study investigated the involvement of ECM-related genes, particularly *THBS1*, in the prognosis and cellular behavior of OSCC. The analysis of ECM-related gene data from OSCC samples identified 165 differentially expressed genes forming two clusters with distinct prognostic outcomes. Seventeen ECM-related genes showed a significant correlation with survival. Experimental methods were employed to demonstrate the impact of *THBS1* on proliferation, migration, invasion, and ECM degradation in OSCC cells. A risk-prediction model utilizing four differentially prognostic genes demonstrated significant predictive value in overall survival. *THBS1* exhibited enrichment of the PI3K/AKT pathway, indicating its potential role in modulating OSCC. In conclusion, this study observed and verified that ECM-related genes, particularly *THBS1*, have the potential to influence the prognosis, biological behavior, and immunotherapy of OSCC. These findings hold significant implications for enhancing survival outcomes and providing guidance for precise treatment of OSCC.

## 1. Introduction

Oral squamous cell carcinoma (OSCC) is the predominant form of head and neck squamous cell carcinoma (HNSCC) that occurs in the oral cavity. It is of great concern globally, ranking as the sixth most commonly diagnosed cancer [1,2]. OSCC is associated with high morbidity and mortality rates, leading to poor long-term patient survival [3]. Although various treatment modalities have been developed for OSCC, such as surgery, chemotherapy, and immunotherapy, their effectiveness in improving patient survival has been limited [4]. Furthermore, these treatments often come with significant side effects, further complicating OSCC patient management [5]. Hence, there is a pressing need for novel therapeutic approaches that can address the limitations of the existing treatment regimens.

The tumor microenvironment (TME) is a complex and interconnected system consisting of multiple components, such as cancer cells, stromal cells, infiltrating immune cells, endothelial cells, lipocytes, and the extracellular matrix (ECM). This intricate network of diverse cellular and non-cellular elements plays a crucial role in tumor development, progression, and response to therapy. The interactions and cross-communication among these components within the TME generate a dynamic environment that can affect tumor behavior and therapeutic outcomes. Gaining a comprehensive understanding of the intricate composition and dynamics of the TME is indispensable for developing efficient strategies to target and manipulate this microenvironment, leading to enhanced cancer treatment approaches [6].

As a crucial component of TME, ECM mainly includes collagen, fibronectin, laminin, glycosaminoglycan, proteoglycan, and various ECM remodeling enzymes [7]. The stiffness and remodeling of ECM structure may play critical roles in tumorigenesis, tumor development, and poor outcomes in a variety of cancers. Tumor development is characterized by complex interactions between cancer cells and the ECM, and the degradation of the ECM is a precondition required for tumor invasion and metastasis [8]. Furthermore, collagen in the ECM is a physical barrier to tumor immune infiltration and an immunosuppressive receptor ligand, and the high density of interconnected collagen fibers within the ECM can block the diffusion of drugs into the tumor [9]. Therefore, exploring the molecular mechanism of ECM degradation in tumor development and therapy is of great clinical significance, especially in OSCC.

Tumor invasion and metastasis are progressive processes that involve several steps: loss of adhesion between tumor cells, formation of adhesion between tumor cells and the matrix or stroma in the TME, changes in the appearance of tumor cells, and formation of invasive synapses known as invadopodia on the surface of cancer cells. These invadopodia can secrete matrix metalloproteinases (MMPs) [10]. The ECM is degraded under the action of matrix metalloproteinases, and finally, tumor cells move and migrate under the induction of some cytokines [11]. It has been shown that cortactin can change the structure of the cytoskeleton and promote the formation of invadopodia, thereby promoting the invasion and metastasis of tumor cells [12]. MMPs can explain almost all the protein components in the ECM and destroy the tissue barrier of tumor cell invasion [11]. In particular, MMP14 can control the formation of invasive pseudopodia [12], while MMP2 and MMP9 play a key role in tumor invasion [13]. Many studies have confirmed that MMP2 and MMP9 are highly expressed in many malignant tumor cells and their stromal components [14,15]. Moreover, MMP2 and MMP9 can be affected by many tumor-related signaling pathways or cytokines [14,15,16].

Cancer immunotherapy has recently emerged as a new approach to cancer treatment, specifically immune checkpoint blockade (ICB) therapy, which has achieved inspiring therapeutic effects in various hematological malignancies [17]. Growing evidence suggests that ICB is more effective against lymphoma than solid tumors, likely due to the physical barriers of interconnected collagen fibers [18,19]. For instance, a study has shown that nintedanib could alleviate collagen fibrosis and distorted vessels in tumor ECM and enhance the efficacy of immune checkpoint blockade (ICB) in murine colon cancer [20]. Recent studies have shown that collagens can affect the function and phenotype of various types of tumor-infiltrating immune cells, such as tumor-associated macrophages (TAMs) and T cells, thus impacting the efficacy of cancer immunotherapy [21,22]. Revealing the molecular mechanism of collagen remodeling in ECM is critical for designing more effective immunotherapy strategies and prognostic signatures for patients with OSCC.

In this study, we classified patients from the TCGA-OSCC cohort into ECM-high and ECM-low subtypes based on the expression of ECM-related genes. We then examined the relationships between clinicopathological features and prognosis in these subtypes. Next, we developed and validated an ECM-related signature (*LAMB4*, *LAMC3*, *PDGFA*, and *THBS1*) specifically for OSCC. Additionally, we modulated *THBS1* expression in OSCC cells to investigate the role of the PI3K/AKT signaling pathway in OSCC cell behaviors and ECM degradation. Overall, our ECM-related signature offers valuable insights for prognostic evaluation and ICB treatment in OSCC.

## 2. Results

### 2.1. Identification of ECM Clusters Based on DEGs Associated with the ECM Scores in OSCC

According to the profiles from the TCGA-OSCC cohort, which consisted of 19 normal samples and 261 OSCC samples, a total of 165 DEGs (141 upregulated genes and 24 downregulated genes) were identified in OSCC samples (Supplementary Figure S1A). Subsequently, these 165 DEGs were uploaded to the Search Tool Retrieval of Interaction Genes (STRING) database to construct a PPI network (Supplementary Figure S1B). The top 30 genes in terms of quantity in the network were as follows: *ITGB1*, *SRC*, *PIK3R1*, *PTK2*, *ITGA1*, *PIK3CA*, *RAC1*, *ITGB3*, *ITGAV*, *RHOA*, *PXN*, *CDC42*, *GRB2*, *SHC1*, *FYN*, *EGFR*, *HRAS*, *BCAR1*, *AKT1*, *ITGA2*, *MET*, *SOS1*, *VEGFA*, *CTNNB1*, *PAK1*, *CRKL*, *FN1*, *LAMA1*, *LAMB1* and *CRK* (Supplementary Figure S1C).

**Figure 1:**
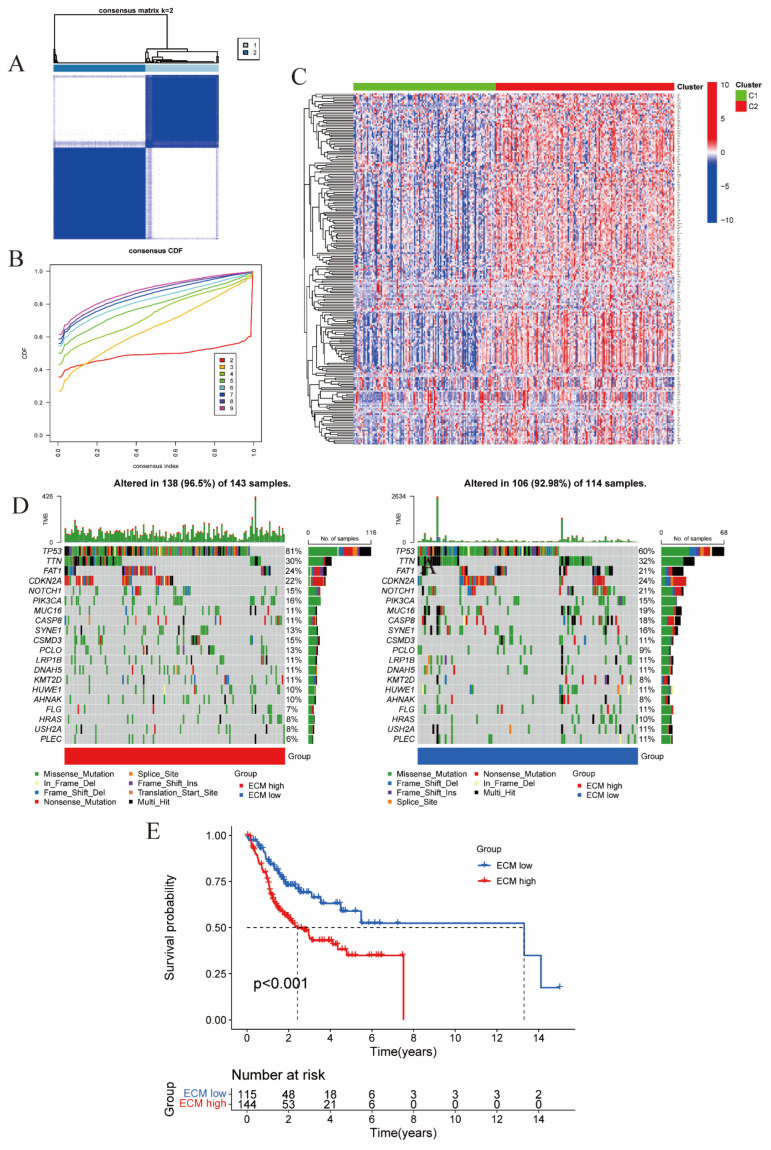
(**A**,**B**) Consensus clustering analysis of ECM-related genes. (**A**) Consensus clustering matrix for k = 2. (**B**) Consensus clustering cumulative distribution function (CDF) for k = 2–9. The area under CDF reached the largest when k = 2. (**C**) Heat map of ECM-related gene expression in clusters C1 and C2. (**D**) The oncoplot of the 20 genes with the highest mutation frequencies in the ECM-high cluster and the ECM-low cluster. (**E**) Kaplan–Meier survival analysis of ECM-high and ECM-low clusters.

**Figure 2:**
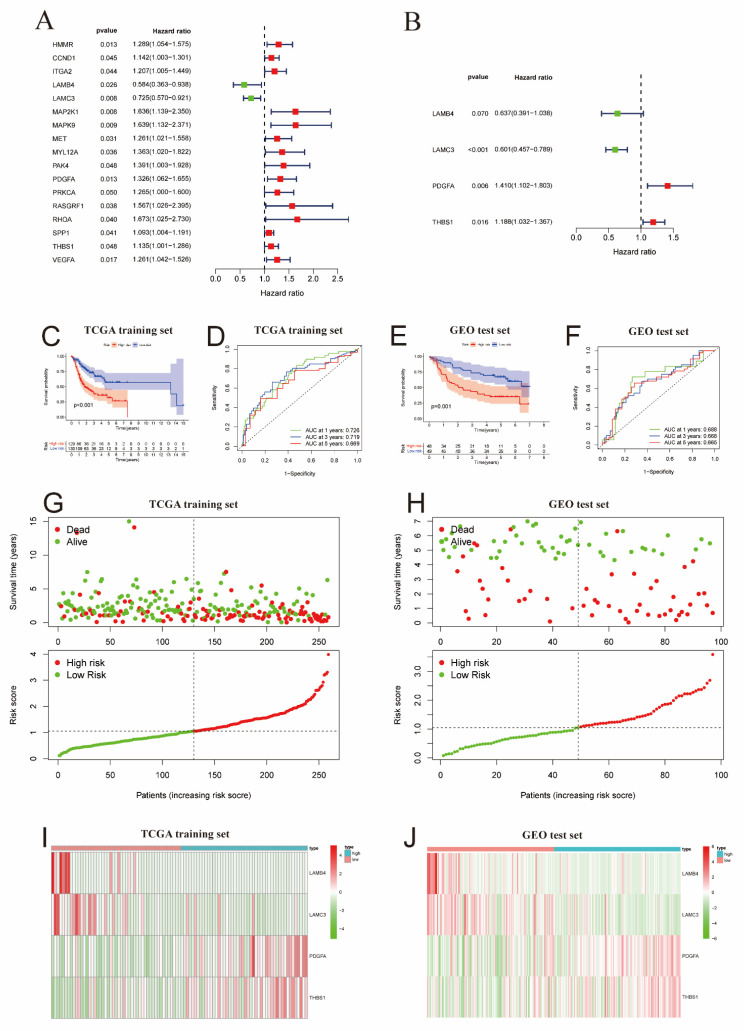
(**A**) Forest plots of 17 ECM-DEGs associated with prognosis by univariate regression analysis. (**B**) Forest plots of the four ECM-DEGs were used to construct the prognostic signature by multivariate Cox regression analysis and stepwise regression analysis. (**C**,**D**) The Kaplan–Meier survival curve and ROC curve were performed in the training set of TCGA. (**E**,**F**) The Kaplan–Meier survival curve and ROC curve were performed in the test set of GEO. (**G**) Reordering of risk scores for each sample in the training cohort and patient survival. (**H**) Reordering of risk scores for each sample in the test cohort and patient survival. (**I**,**J**) Heat map of four prognosis-related ECM-DEG expression profiles in the training cohort (**I**) and the test cohort (**J**) in the low- and high-risk groups.

The collected 261 OSCC samples were subjected to cluster analysis based on the expression of ECM-DEGs. Clustering results for k values ranging from 2 to 9 revealed that the samples showed optimal clustering when divided into two clusters: Cluster 1 (ECM-high) with 156 samples and Cluster 2 (ECM-low) with 105 samples. This division was supported by a consensus matrix displaying a relatively uniform distribution, minimal overlap, and a progressively decreasing CDF value (Figure 1A,B). Consequently, the OSCC samples were categorized into ECM-high and ECM-low clusters. To visualize gene mutations, we analyzed data from 257 TCGA-OSCC samples and identified the 20 genes with the highest mutation frequencies across the entire sample set, representing the ECM-high and ECM-low groups (Figure 1C). Among these highly mutated genes, seven were upregulated in the ECM-high group compared with the ECM-low group: *TP53*, *FAT1*, *PIK3CA*, *PCLO*, *KMT2D*, *CSMD3*, and *AHNAK* (Figure 2C, left panel). Conversely, eleven genes were downregulated in the ECM-high group: *TTN*, *CDKN2A*, *NOTCH1*, *MUC16*, *CASP8*, *SYNE1*, *HUWE1*, *FLG*, *HRAS*, *USH2A*, and *PLEC* (Figure 1C). Additionally, we performed outcome survival (OS) analysis on clinical survival data for the ECM-high and ECM-low samples. Kaplan–Meier curve analysis demonstrated a significantly improved clinical prognosis for OSCC patients in the ECM-low group compared with the ECM-high group (Figure 1D).

### 2.2. Characteristics of Immune Infiltration in Different ECM Clusters of OSCC

To investigate the underlying mechanism responsible for prognostic differences among different extracellular matrix (ECM) clusters, we analyzed differentially expressed genes (DEGs) between the OSCC ECM-high and ECM-low groups. DEGs were determined using a cutoff criterion of log2|FC| > 1 and FDR < 0.05, resulting in a total of 1519 ECM-DEGs (Supplementary Figure S2A). We subsequently conducted a functional enrichment analysis of ECM-DEGs and identified significant differences in both ECM-associated pathways and immune-related pathways between the two ECM clusters. The results of the GO analysis revealed a significant enrichment of ECM-DEGs in biological processes (BPs) related to cell chemotaxis (Supplementary Figure S2B). Furthermore, GSEA (gene set enrichment analysis) was employed to validate the aforementioned results, demonstrating a significant enrichment of ECM-related pathways in the ECM-high group (Supplementary Figure S2C). Conversely, the immune response in the ECM-low group exhibited a significant increase (Supplementary Figure S2D). We subsequently analyzed the immune characteristics (stromal scores, estimate scores, immune scores, and tumor purity) of the OSCC ECM-high and ECM-low clusters using the ESTIMATE algorithm. Our findings revealed that the stromal and ESTIMATE scores in the ECM-high group were higher compared with the ECM-low clusters, while the tumor purity was lower in the ECM-high group (Supplementary Figure S2E–H). We then conducted an exploration of the proportional histogram and correlation among different types of immune cells in OSCC samples from the TCGA dataset. Our investigation revealed significant changes in the internal proportions of immune cells between the two clusters (Supplementary Figure S2I). In comparison to the ECM-low group, we observed an increase in the proportions of plasma cells, resting CD4 T cells, M2 macrophages, and activated mast cells, while the proportions of memory B cells, CD8 T cells, activated CD4 T cells, Tfh cells, Tregs, activated NK cells, monocytes, M0 macrophages, and resting mast cells decreased (Supplementary Figure S2J). These results indicate a strong correlation between the expression levels of ECM-related genes and the TME in OSCC.

### 2.3. Development and Verification of the ECM Prognostic Signature for OSCC

Univariate Cox regression analysis was performed on 215 ECM-related genes and we identified 17 ECM-related genes that showed potential as prognostic markers (Figure 2A). We came to similar conclusions, through multivariate Cox regression analysis and stepwise regression analysis of the training set samples from the TCGA database. Subsequently, we selected four genes related to prognosis to construct an ECM prognostic risk signature (referred to as risk scores) (Figure 2B). We calculated the *ECM risk scores* for each case using the following formula: risk scores = *LAMB4* × (−0.4507) + *LAMC3* × (−0.5095) + *PDGFA* × 0.3434 + *THBS1* × 0.1720. OSCC patients in the training and test sets were categorized into high-risk and low-risk groups based on the median value of the risk score (1.055).

We analyzed the predictive capability of the ECM signature using outcome survival (OS) analysis in both the training and test sets. In the training subset, the low-risk subgroup exhibited significantly better clinical outcomes compared with the high-risk subgroup (Figure 2C). The area under the curve (AUC) of the matrix risk score was 0.726, 0.719, and 0.669 at 1, 3, and 5 years, respectively, as determined by ROC curve analysis (Figure 2E). Kaplan–Meier curves in the independent external validation test subset GSE41613 demonstrated that the ECM signature effectively classified cases into subgroups with distinct prognoses (Figure 2D). The ROC curves also demonstrated strong predictive capacity of the matrix risk score at 1, 3, and 5 years (Figure 2F). In the risk-distribution and survival-status pattern plot, the majority of patients in the training group and the high-risk group experienced mortality, whereas the majority of patients in the low-risk subgroup survived (Figure 2G), and the same results were found in the GEO test subset (Figure 2H). The heat map displaying the expression patterns in the high-risk and low-risk groups revealed that OSCC patients with high levels of *PDGFA* and *THBS1* expression had shorter survival time and a worse clinical prognosis, while patients with high levels of *LAMB4* and *LAMC3* expression had a longer survival time and a better clinical prognosis (Figure 2I,J). We conducted independent prognostic analysis on various clinical indicators and the ECM signature of OSCC patients using univariate Cox regression and multivariate Cox regression. The results showed that the ECM signature we developed functioned as an independent prognostic factor, unaffected by other factors (Supplementary Figure S3A,B), and the same results were observed in the GEO test subset (Supplementary Figure S3C,D).

### 2.4. Correlation Analysis of ECM Risk Scores and Immune Infiltration in OSCC

To investigate the association between the extracellular matrix (ECM) signature and intra-tumoral immune infiltration characteristics, we utilized the CIBERSORT algorithm to determine the proportional representation of 15 immune-cell types in a cohort of patients with oral squamous cell carcinoma (OSCC). Our analysis revealed a significant correlation between the risk scores and specific immune-cell populations. CD8+ T cells, T-regulatory cells (T-regs), T cells follicular helper (Tfh) cells, and naive B cells exhibited a negative correlation with risk scores (Supplementary Figure S4A–D). Conversely, resting natural killer (NK) cells and resting CD4 memory T cells demonstrated a positive correlation with risk scores (Supplementary Figure S4E,F).

### 2.5. Ectopic Expression of THBS1 Is Correlated with Clinical Parameters of OSCC

Previous studies have established a significant association between *THBS1* and prognosis as well as the tumor microenvironment in patients with oral squamous cell carcinoma (OSCC). These findings prompted us to investigate whether *THBS1* may serve as a promising prognostic factor and oncogene for OSCC patients. Initially, we analyzed the expression of *THBS1* in OSCC tissues and normal tissues using the TCGA and GEO databases. Interestingly, the results revealed a significant upregulation of *THBS1* in OSCC (Supplementary Figure S5A). Moreover, we examined the prognostic significance of *THBS1* in OSCC patients through the KM-plot database, which indicated a strong inverse correlation between *THBS1* expression levels and OS (Supplementary Figure S5B). To visualize *THBS1* expression patterns, we retrieved immunohistochemical maps from The Human Protein Atlas (https://www.proteinatlas.org, accessed on 1 January 2023) for both normal samples and OSCC tumor tissues. Consistently, our analysis demonstrated a significantly higher expression of *THBS1* in OSCC samples compared with normal tissues (Supplementary Figure S5C). Additionally, the gene set variation analysis (GSVA) heatmap revealed several pathways associated with *THBS1* expression in OSCC. Notably, elevated *THBS1* levels were closely linked to cell-cycle regulation, DNA replication, and proteasome processes. Conversely, reduced *THBS1* levels were associated with immune-related pathways, including natural-killer-cell differentiation, leukocyte proliferation, and immune response (Supplementary Figure S5D). These collective findings strongly suggest that *THBS1* may play a pivotal role in the development and progression of OSCC.

### 2.6. THBS1 Can Over-Activate the PI3K/AKT Signaling Pathway in OSCC

**Figure 3:**
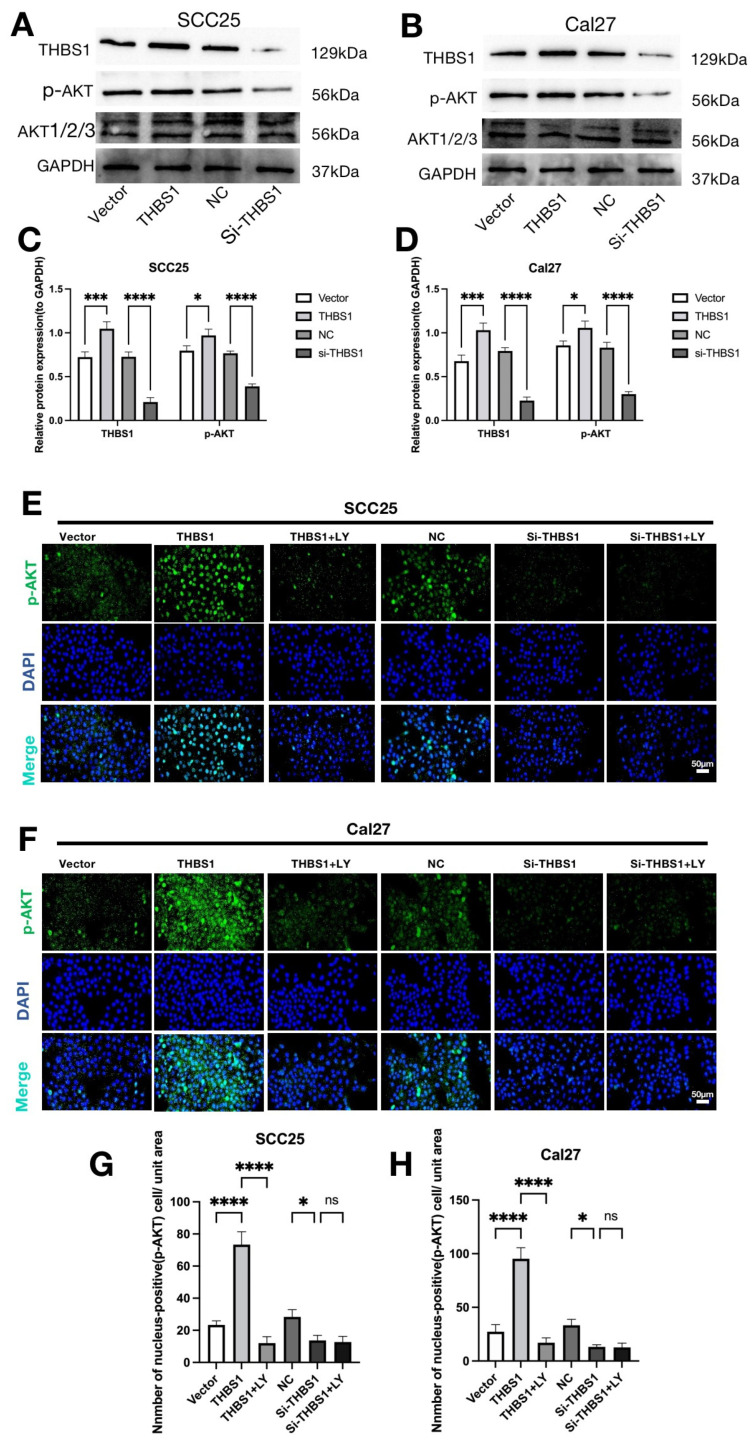
(**A**,**B**) WB was used to detect the activation of the PI3K/AKT signaling pathway after overexpression or knockdown of *THBS1* in two OSCC cell lines. (**C**,**D**) Quantitative analysis of p-AKT protein expression levels in OSCC cells (*n* = 3, one-way ANOVA followed by Tukey’s multiple comparisons; vector vs. *THBS1* and NC vs. si-*THBS1*, * *p* < 0.05, *** *p* < 0.001, **** *p* < 0.0001). (**E**,**F**) Immunofluorescence was used to detect the expression and nuclear translocation of p-AKT in OSCC cells after overexpression or knockdown of *THBS1* or treatment with 10 nM LY294002 inhibitor (scale bar, 50 μm). (**G**,**H**) Quantitative analysis of p-AKT nuclear expression in two OSCC cells (*n* = 3, one-way ANOVA followed by Tukey’s multiple comparisons; vector vs. *THBS1* and NC vs. si-*THBS1*, * *p* < 0.05, **** *p* < 0.0001), ns: no significant.

Based on the above data analysis of *THBS1*, we investigated the over-expression and knockdown of *THBS1* in OSCC (SCC25 and Cal27) cells to observe the effect of *THBS1* expression on the malignant biological behavior of OSCC cells. First, we used the *THBS1* plasmid and two different siRNA to construct *THBS1* overexpression or knockdown transient transfected OSCC cells in two OSCC cell lines, and WB was used to verify the results, as shown in Supplementary Figure S3E,F. The results showed that si2-*THBS1* knockdown efficiency was high. Therefore, si2-*THBS1* was selected for subsequent experimental study. WB results showed (Figure 3A–D) that the overexpression of *THBS1* in OSCC could over-activate the PI3K/AKT signaling pathway, mainly manifested as the activation of AKT phosphorylation site Ser473. Conversely, the knockdown of *THBS1* resulted in the inhibition of the PI3K/AKT signaling pathway, this was consistent with our results of significant enrichment of the PI3K/AKT signaling pathway in the *THBS1* high expression group in Supplementary Figure S5D.

To validate that the PI3K/AKT signaling pathway is primarily regulated by *THBS1*, we treated OSCC cells with the PI3K/AKT signaling pathway inhibitor (LY294002) and observed the nuclear translocation of p-AKT. The results of IF showed that p-AKT was highly expressed in the cytoplasm and nucleus after overexpression of *THBS1*, as indicated by the prominent green fluorescence. However, when LY294002 was administered following *THBS1* overexpression, the nuclear expression of p-AKT in OSCC cells dramatically decreased. Similar observations were identified in the knockdown group, where the nuclear expression of p-AKT significantly decreased upon *THBS1* knockdown. Notably, when *THBS1* knockdown was combined with LY294002 treatment, there was no significant difference in the nuclear changes of p-AKT compared to *THBS1* knockdown alone (Figure 3E–H). This suggests that *THBS1* primarily functions as a mediator of the PI3K/AKT signaling pathway in OSCC cells.

### 2.7. THBS1 Mediates Cell Proliferation via the PI3K/AKT Signaling Pathway in OSCC

**Figure 4:**
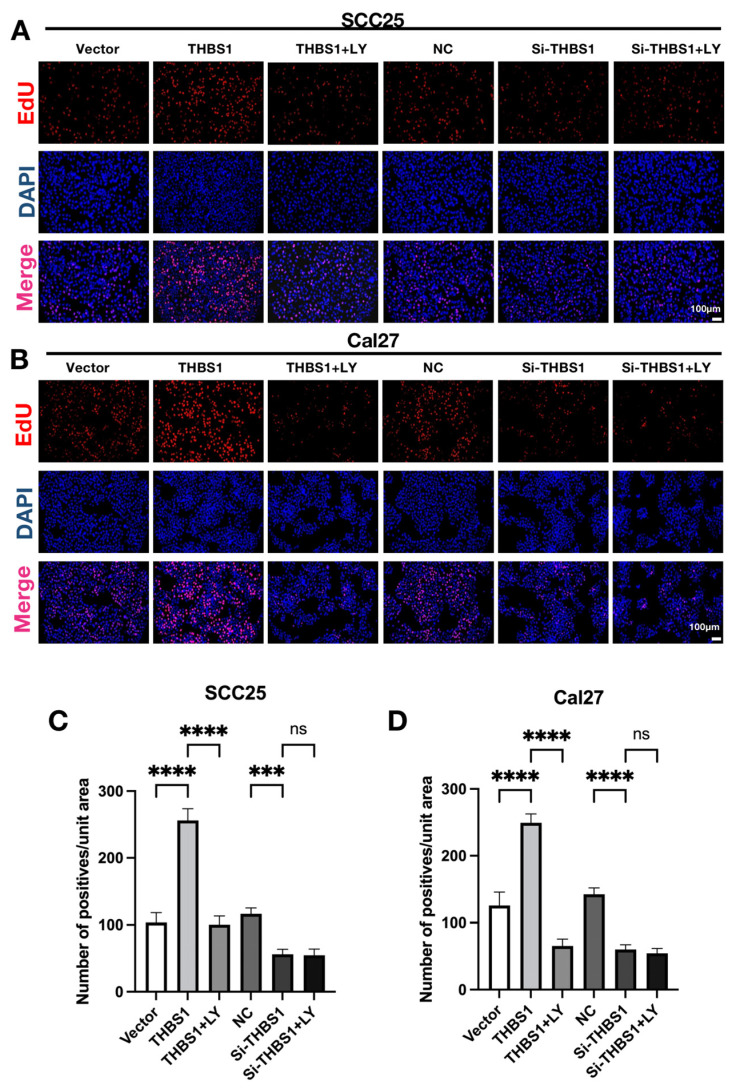
(**A**,**B**) The proliferation of OSCC cells was detected by EdU assays at 48 h after transfection and treatment with 10nM LY294002 (scale bar, 100 μm). (**C**,**D**) Quantitative analysis of the number of EdU positives (unit area = 850 μm × 500 μm) (*n* = 3, one-way ANOVA followed by Tukey’s multiple comparisons; vector vs. *THBS1*, *THBS1* vs. *THBS1* + LY294002, NC vs. si-*THBS1*, and si-*THBS1* vs. si-*THBS1* + LY294002, *** *p* < 0.001, **** *p* < 0.0001).

To explore whether *THBS1* could promote the proliferation of OSCC cells by mediating the PI3K/AKT signaling pathway, we used an EdU cell proliferation kit to detect the proliferation of OSCC cells (Figure 4A–D). We found that the number of proliferations (red fluorescence) of OSCC cells after overexpression of *THBS1* was significantly more than that of the vector group. On the contrary, the number of proliferations of OSCC cells after the knockdown of *THBS1* was remarkably less than that of the NC group (Figure 4A–D). Interestingly, treatment with LY294002 in combination with *THBS1* overexpression significantly reduced the number of proliferating cells, while *THBS1* knockdown combined with LY294002 treatment had no significant effect on cell proliferation compared with *THBS1* knockdown alone. WB also obtained similar results. As shown in Figure 4E,F, the proliferation antigen PCNA was regulated by the expression of *THBS1*, which was consistent with the expression trend of *THBS1*. When *THBS1* was overexpressed or knocked down, the cells were treated with LY294002. The results showed that LY294002 could attenuate the proliferation effect of the *THBS1* group, while there was no obvious difference in the trend of LY294002 treatment after *THBS1* knockdown. These results indicated that *THBS1* could regulate the proliferation of OSCC cells, and suggested that *THBS1* mainly mediated the PI3K/AKT signaling pathway.

### 2.8. THBS1 Mediates the Migration and Invasion Behaviors via the PI3K/AKT Signaling Pathway in OSCC

**Figure 5:**
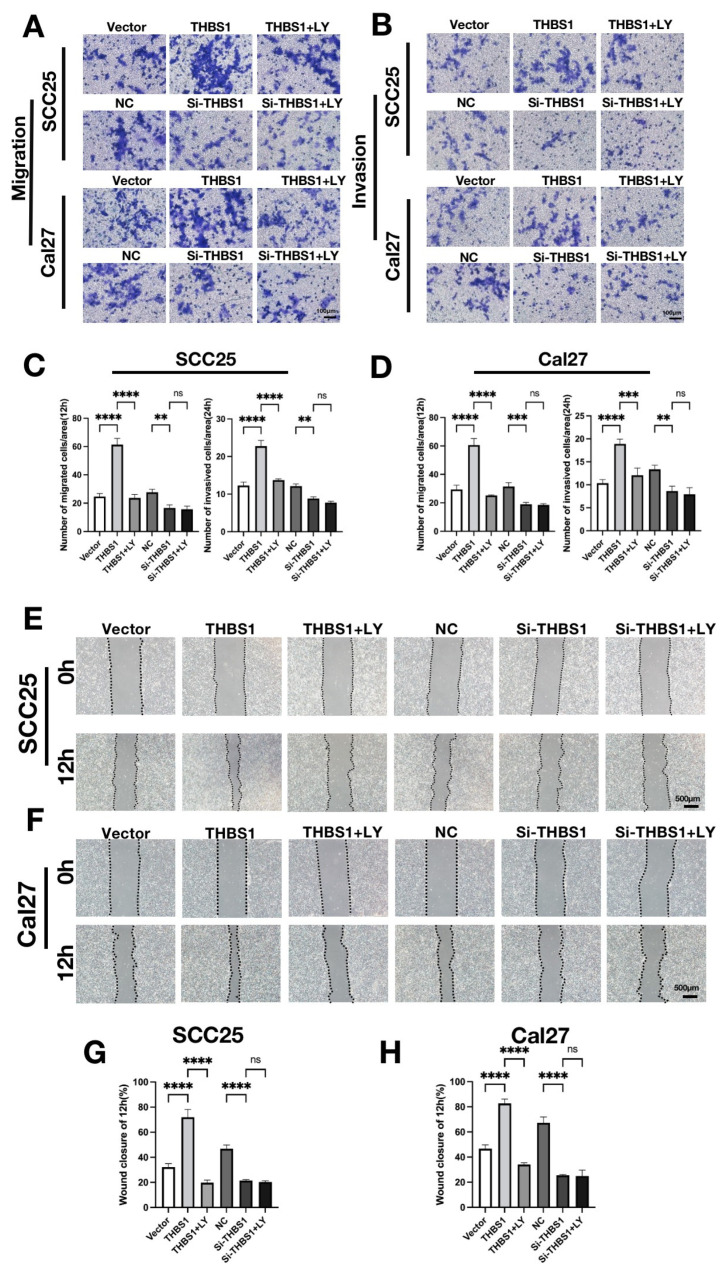
(**A**,**B**) The migration and invasion abilities were detected by Transwell assays after OSCC cells were treated with transfection or inhibitor for 48 h (scale bar, 100 μm). (**C**,**D**) Quantitative analysis of the number of migrated and invasive cells in (**C**,**D**) (*n* = 3, one-way ANOVA followed by Tukey’s multiple comparisons; vector vs. *THBS1*, *THBS1* vs. *THBS1* + LY294002, NC vs. si-*THBS1*, and si-*THBS1* vs. si-*THBS1* + LY294002, ** *p* < 0.01, *** *p* < 0.001, **** *p* < 0.0001). (**E**,**F**) The wound healing assay was used to detect the horizontal migration ability of OSCC cells 48 h after transfection and after inhibitor treatment (scale bar, 500 μm). (**G**,**H**) The wound closure area of OSCC cells in (**E**,**H**) was quantitatively analyzed (*n* = 3, one-way ANOVA followed by Tukey’s multiple comparisons; vector vs. *THBS1*, *THBS1* vs. *THBS1* + LY294002, NC vs. si-*THBS1*, and si-*THBS1* vs. si-*THBS1* + LY294002, **** *p*< 0.0001).

Furthermore, we investigated whether *THBS1* mediated the PI3K/AKT signaling pathway in OSCC cell migration and invasion. Cell migration was initially assessed through a wound-healing assay (Figure 5E–H) and Transwell migration assay (Figure 5A,C). The findings revealed that cells in the *THBS1* group exhibited a higher migration rate compared with the vector group. However, the migration ability was hindered by the LY294002 treatment. Similarly, the migration rate of Si-*THBS1* cells was significantly lower than that of the negative control group. Notably, knocking down *THBS1* and subsequently adding LY294002 did not further reduce the migration of OSCC cells (Figure 5A,C). Overall, these results provide substantial evidence that the *THBS1* pathway mediates the PI3K/AKT signaling pathway in regulating the migration ability of OSCC.

**Figure 6:**
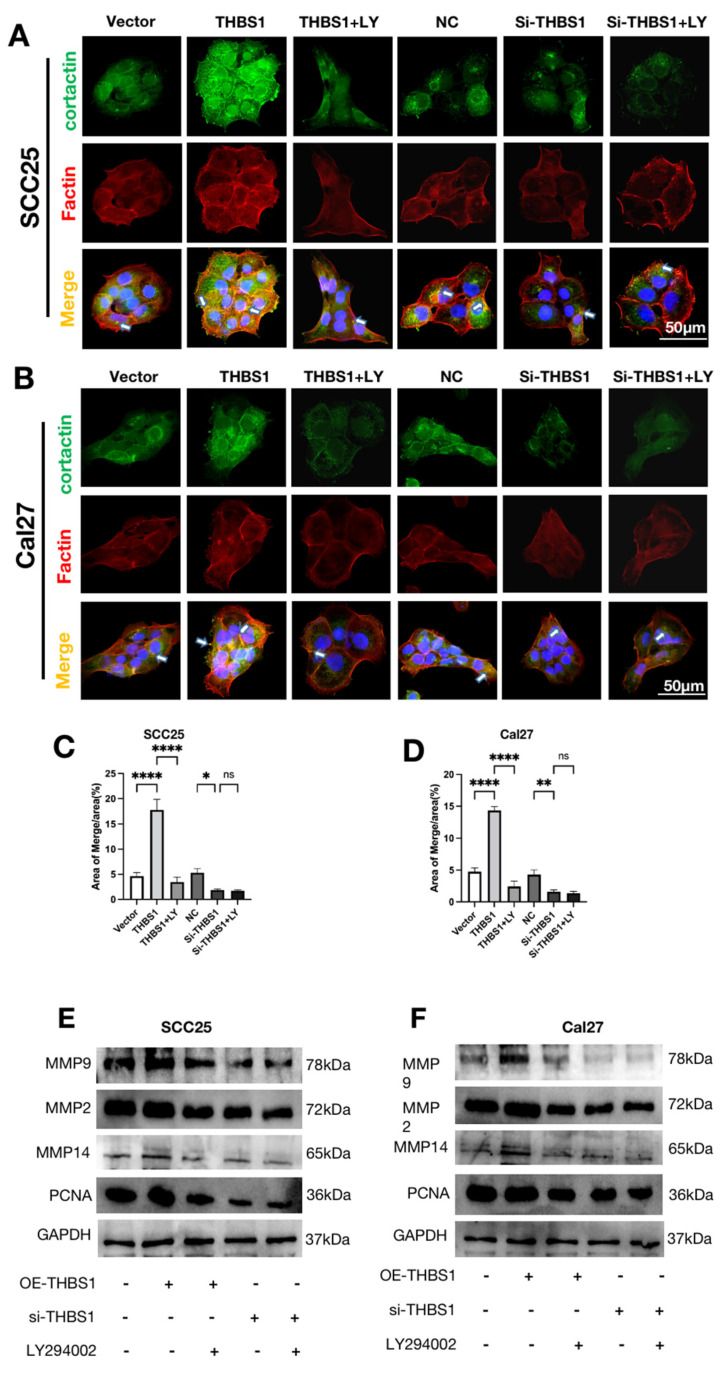
(**A**,**B**) Immunofluorescence double staining of cortactin and F actin was performed to detect invadopodia in OSCC cells after transfection or treatment with 10nM LY294002. Red fluorescence represents F actin, green fluorescence represents cortactin, and the merged images (yellow) show colocalization of F actin (red) and cortactin (green), indicating invadopodia formation (scale bar, 50 μm). (**C**,**D**) Quantitative analysis of the area of invadopodia (yellow) per cell (*n* = 3, one-way ANOVA followed by Tukey’s multiple comparisons; vector vs. *THBS1*, *THBS1* vs. *THBS1* + LY294002, NC vs. si-*THBS1*, and si-*THBS1* vs. si-*THBS1* + LY294002, * *p* < 0.05, ** *p* < 0.01, **** *p* < 0.0001). (**E**,**F**) WB was used to detect the expressions of MMP2, MMP9, MMP14, and PCNA in OSCC cells treated with transfection or inhibitor.

To comprehend the mechanism by which *THBS1* facilitates extracellular matrix degradation in OSCC, we employed the Transwell invasion assay (Figure 5B,D) and invadopodia fluorescence co-localization staining to observe this phenomenon (Figure 6A–D). The invasion results showed that the number of cells in the *THBS1*-overexpression group was increased, while the number of cells in the Si-*THBS1* group was significantly decreased. The addition of LY294002 to the *THBS1* group led to a sharp decrease in the number of cells in the permeable chamber. However, the addition of LY294002 to the knockdown group did not significantly alter the results (Figure 5B,D). These findings confirm that *THBS1* primarily mediates the PI3K/AKT signaling pathway to regulate invasion in OSCC.

### 2.9. THBS1 Promotes Invadopodia Formation and ECM Degradation through PI3K/AKT Signaling Pathway

Invadopodia, characterized by co-localization of cortactin and F-actin, and high MMP14 expression in invadopodia are essential for tumor cell invasion. Prior studies have highlighted the role of invadopodia in extracellular matrix degradation through the secretion of two pivotal matrix metalloproteinases (MMPs), MMP2 and MMP9. Therefore, it is of particular importance to assess the expression of invadopodia in OSCC. Our investigation demonstrated that *THBS1* can regulate the generation of invadopodia. Overexpression of *THBS1* resulted in an increased number of invadopodia, while knockdown of *THBS1* exhibited the opposite effect. This regulation occurred through the PI3K/AKT signaling pathway (Figure 6A–D). The *THBS1* group displayed the highest expression levels of MMP2, MMP9, and MMP14 among the two OSCC cell lines, whereas LY294002 successfully reversed the expression of MMP2, MMP9, and MMP14 in the *THBS1* group. The si-*THBS1* group and si-*THBS1* + LY294002 group demonstrated lower expressions of MMP2 and MMP9 and MMP14, with no significant differences between them (Figure 6E,F).

## 3. Discussion

ECM components provide cells with both biochemical and biomechanical support [23]. The highly interconnected and dynamically arranged macromolecule that forms the ECM is critical to cellular behavior, with the structure of the components and their associations with each other have heterogeneous effects [24]. The importance of the ECM in cancer progression and metastasis has led to increased attention, with many studies exploring the dysregulated expression of ECM-related genes in various cancers [7,23,24,25,26]. However, no comprehensive analysis of the entire ECM genome in OSCC has been conducted. Therefore, we performed the first systematic analysis of ECM-related genes, examining their potential functions and prognostic value in OSCC using TCGA and GEO datasets.

In this study, we first identified DEGs in normal and OSCC tumor samples then examined the protein interaction network of DEGs and discovered 30 key genes. Then, based on the level of ECM gene expression, we used unsupervised hierarchical clustering to classify OSCC tumor samples into ECM-high and ECM-low groups. Recently, several TMEs, including pancreatic ductal adenocarcinoma, colorectal cancer, and OSCC, have used the ESTIMATE algorithm to derive immunologic, stromal, and immune scores [27,28,29]. In this work, we used ESTIMATE to discover that even though the expression levels of ECM-related genes did not affect the overall proportion of immune cells in the TME in OSCC patient samples, the composition of immune cells varied dramatically. Notably, in the ECM-high group, CD8 T cells were dramatically downregulated, which may impair patients’ ability to mount an effective anti-tumor immune response and worsen prognosis. The characteristics of the TME affect the ability of tumor-specific T cells to inhibit tumor growth, and the composition of the ECM can directly influence tumor growth as well as T-cell infiltration and function [30,31,32]. Enhancing the adaptive immune response by altering the nature of the ECM to induce processes that ultimately lead to tumor regression may be a promising therapeutic approach.

Intriguingly, the ECM-high group’s immunoglobulin complex and receptor functional signals were decreased according to the GSEA functional enrichment results, indicating that this group’s immune response to cancer may be impaired. Furthermore, the results of the GSEA pathway enrichment revealed that the cytochrome-P450, linoleic-acid, and arachidonic-acid metabolic pathway signals were reduced in the ECM-high group. An immunosuppressive tumor microenvironment caused by high amounts of arachidonic acid was discovered in earlier research, which was demonstrated by arachidonic acid’s decreased transcriptional responses to interferon and IL-6 in monocyte-derived macrophages [33]. Recently, linoleic acid has been shown to be a positive regulator of CD8+ T-cell activity, improving T-cell metabolic capacity, preventing T-cell depletion, and stimulating T-cell differentiation toward a powerful effector memory-like phenotype with enhanced antitumor effects [34]. *CYP1B1* is a member of the cytochrome P450 family, and CYP1B1 protein is highly expressed in most human tumor tissues but is low or not expressed in tumor-free tissues [35,36]. Several drugs characterized by this specific metabolic activation have been developed and are in preclinical evaluation [35,36,37].

To effectively predict the prognosis of OSCC patients, we initially identified 17 candidate ECM-related genes that are related to the prognosis. Next, we developed a prognostic signature, referred to as the matrix risk score, using four ECM-related genes (*PDGFA*, *THBS1*, *LAMB4*, and *LAMC3*) in the training set. We then verified the effectiveness of this signature using the test and validation sets. The matrix risk score indicates that high expression levels of *PDGFA* and *THBS1* are risk factors, whereas increased expression levels of *LAMB4* and *LAMC3* offer a protective effect. The platelet-derived growth factor (PDGF) family plays a crucial role in various biological processes associated with malignant growth, such as angiogenesis, fibrosis, and cell migration, through paracrine interactions. PDGFs have also been shown to mediate oncogenic signaling in an autocrine manner in multiple malignancies [38]. *PDGFA* has been found to have a pivotal regulatory function in several cancers, such as head and neck squamous cell carcinoma, pancreatic ductal adenocarcinoma, and papillary thyroid carcinoma [38,39,40,41,42]. Additionally, thrombospondin 1 (*THBS1*), an oncogene in OSCC, is a tumor-specific ECM protein that is induced by *TGFB1* and promotes cancer-cell migration while stimulating MMPs partly through integrin signaling expression, which facilitates the invasion of OSCC [43,44,45]. Moreover, the expression of *THBS1* is closely related to the proliferation of cancer cells. After knocking down *THBS1* in laryngeal cancer and nasopharyngeal cancer, CCK8 detected that the proliferation activity of laryngeal cancer cells was significantly downregulated [46]. Secondly, numerous studies have demonstrated that *THBS1* can excessively activate the PI3K/AKT signaling pathway and enhances cell viability [47]. Moreover, activated AKT directly phosphorylates precursor apoptotic proteins, such as Bad, resulting in immediate effects that hinder the activation of the apoptotic pathway and consequently lead to cell death. At the same time, MMP2 and MMP9, and other MMPs can be regulated by activated AKT, and tumor cells with excessive activation of the PI3K/AKT signaling axis are more likely to undergo EMT [48,49]. The activation of AKT may be one of the key processes of tumor migration and invasion. *THBS1* provides strong survival ability, migration, and invasion of tumor cells by mediating excessive activation of the PI3K/AKT signaling pathway [46].

In our study, we overexpressed and knocked down the *THBS1* gene in OSCC cells, and found that the malignant biological behavior of OSCC cells was significantly different with the change of *THBS1*. Overexpression of *THBS1* could increase the proliferation, migration, and invasion ability of OSCC cells, promote the formation of invadopodia, and degrade the ECM. After *THBS1* knockdown, the proliferation, migration, and invasion of OSCC cells were significantly inhibited, and the formation of invadopodia was significantly inhibited to prevent ECM degradation. Invadopodia are membrane protuberous structures rich in filamentous actin formed on the membrane of tumor cells. Actin rearrangement changes the original cell morphology and adhesion ability, which is a necessary condition for tumor invasion and metastasis, and MMP14, as well as cortactin, plays a key role in the formation of invadopodia [12]. After signal pathway enrichment analysis, we found that *THBS1* could also significantly activate the PI3K/AKT signaling pathway in OSCC. WB and IF assay detection showed that *THBS1* could phosphorylate Ser473 on AKT protein, and the phosphorylation at the Ser473 site could make AKT exert full enzymatic activity [50]. Interestingly, LY294002 significantly reversed the proliferation, migration, invasion, and invadopodia formation of OSCC cells overexpressing *THBS1*. However, the malignant biological behavior of OSCC cells treated with LY294002 in the knockdown group did not change significantly, which confirmed that *THBS1* can mediate the PI3K/AKT signaling pathway to regulate the malignant biological behavior of OSCC.

In addition, a previous study investigating the effect of conventional chemotherapy on tumor angiogenesis in breast cancer found that increased *VEGFA* expression correlated with tumor recurrence. Additionally, some patients with higher levels of *THBS1* showed increased angiogenic responses following chemotherapy [51]. Previous research has shown that gene mutations in certain patients with gastric cancer, colorectal cancer, and pancreatic cancer can cause a decrease or absence of laminin β4 (*LAMB4*) expression [52,53]. Furthermore, in non-muscle invasive bladder cancer, colorectal cancer, and breast cancers, high levels of laminin γ3 (*LAMC3*) expression have been linked to a positive prognosis [54,55,56].

Currently, chemotherapy and immunotherapy remain key strategies in the multimodal treatment of advanced OSCC, with some patients achieving stable responses to immunotherapy [57,58]. However, despite these treatments, the prognosis for advanced OSCC has not improved significantly, and only a small fraction of patients appear to benefit from immunotherapy [58,59]. To address this issue, we have developed a unique gene signature consisting of four extracellular matrix-related genes that may be useful in predicting the prognosis of OSCC patients and evaluating tumor immunity. This information could potentially facilitate the development of personalized treatment approaches for OSCC patients. However, our study still had limitations. Whether key ECM markers derived from stromal markers can also be assessed at the protein level by immunohistochemistry for prognosis (risk signature) of premalignant lesions, and for OSCC diagnosis (stromal typing as ECM-high or ECM-low), remains to be determined. Moreover, our study was retrospective. Prospective clinical and mechanistic studies are needed for further validation of our findings.

## 4. Materials and Methods

### 4.1. Data Collection and Processing

Gene expression, somatic mutation, and corresponding clinicopathological data of 280 OSCC samples (261 tumor samples, and 19 normal samples, accessed on 1 January 2023) were obtained from the TCGA database and comprised samples from various areas within the oral cavity, including the floor of the mouth, the base of the tongue, the lip, the palate, and other unspecified parts of the tongue and mouth) were obtained from the TCGA database (https://portal.gdc.cancer.gov/, accessed on 1 January 2023) in March 2023. Cases with prognostic information were used as the training set. As the external validation set, 97 microarrays and clinical data for OSCC from GSE41613 were downloaded from the GEO database (https://www.ncbi.nlm.nih.gov/, accessed on 1 January 2023). A total of 215 ECM-associated genes were downloaded from two ECM-associated gene sets (“KEGG ECM RECEPTOR INTERACTION” and “KEGG FOCAL ADHESION”) of the GSEA Molecular Signatures Database (https://www.gsea-msigdb.org/gsea/msigdb/index.jsp, accessed on 1 January 2023).

### 4.2. Identification of ECM-Related DGEs (Differentially Expressed Genes)

The “Limma” R package was utilized for conducting differential gene expression analysis between tumor and non-tumor tissues [60]. A significance level of *p* < 0.05 was utilized to determine the significance of gene expression differences between OSCC and adjacent normal tissue. Gene expression differences between the ECM-high and ECM-low subgroups were considered significant if *p* < 0.05 and logFC > 1. Heatmaps of gene expression matrices were constructed using the “ComplexHeatmap” R package for visualization purposes [61].

### 4.3. Tumor Sample Clustering

The tumor samples obtained from the TCGA database were separated into ECM-high and ECM-low groups using K-means clustering, based on the expression levels of ECM genes, implemented through the “Consensus Cluster Plus” R package [62].

### 4.4. Analysis of Tumor Gene Mutations

Mutations within the ECM-high and ECM-low groups were analyzed and visualized using the R package “maptools” [63]. Subsequently, a comparison was performed between these two subgroups to assess the potential correlation between ECM and tumor gene mutations.

### 4.5. Development and Validation of ECM-Related Signature for OSCC

To identify potential predictive genes, a univariate Cox regression analysis was conducted. Subsequently, the risk model was constructed through stepwise regression analysis in the training set. The candidate genes were then subjected to multifactorial Cox regression analysis using the R programming language [64]. Matrix risk score, which was used to determine the risk score for each clinical case, was computed using the following formula:

(1) $$ECM\;Risk\;Score=\sum_{i=1}^{n}\beta_{i}S_{i}$$

where i = gene in the matrix risk signature of length *n*, ${\;S}_{i}\;$= S-scaled gene expression of gene I, and $\beta_{i}$ = log (odds ratio) of gene *i.*

Following that, the cases within both the training and test sets were divided into two subgroups, namely high-risk and low-risk, based on the median value of the matrix risk core.

### 4.6. Functional and Pathway Enrichment Analysis

We employed the R package “clusterProfiler” to perform gene set enrichment analysis (GSEA) and explore the potential functions and pathways associated with the ECM-high and ECM-low subgroups [65,66]. A significance level of *p* < 0.05 was employed to assess the statistical significance of enriched groups, and the resulting enrichment graph was generated using the R package “enrich plot”.

### 4.7. Immune Infiltration Study of OSCC

We estimated the immune scores using the “ESTIMATE” R package and assessed tumor infiltration using the “CIBERSORT” R package. The ESTIMATE and CIBERSORT algorithms were employed to investigate the association between subgroups and tumor immunity [67,68]. We studied the variations in immunological ratings and the infiltration of immune cells in tumors among the ECM-high and ECM-low subgroups, as well as the high-risk and low-risk groups. To analyze the correlation between immunological score and ECM, we performed a correlation analysis. The submap algorithm was used to evaluate the response of OSCC to anti-PD1 and anti-CTLA4 therapy. The TIDE algorithm was employed to assess the response to anti-immune checkpoint therapy.

### 4.8. Cell Culture, Cell Transfection, and Reagents

The SCC25 and Cal27 cells were obtained from the China Center for Type Culture Collection in Shanghai, China. The cells were cultured in DMEM/F12 culture medium supplemented with 10% fetal bovine serum (FBS; BI, Biological Industries; Shanghai, China) and 100 U/mL penicillin and streptomycin (BI) at 37 °C in a humidified atmosphere with 5% CO_2_. The pcDNA3.1 vector and *THBS1* plasmid, as well as negative control and small interfering RNA (siRNA), were purchased from GenePharma Co., Limited in Shanghai, China. For cell inoculation, 1 × 10^5^ cells were seeded into 6-well plates and cultured until they reached 60–90% confluency. Subsequently, cell transfection was carried out using Beyotime (Shanghai, China) Lipo8000TM transfection reagent. The transfection mixture, consisting of 2500 ng plasmid or 37.5 pmol siRNA, was combined with 4 μL of Lipo8000 and incubated at 37 °C. Experimental treatments were conducted 48 h after transfection. Commonly used reagents included: PI3K/AKT signaling pathway inhibitor LY294002 (Beyotime, Shanghai, China), 1% crystal violet staining solution (Coolaber, Beijing, China), and the Easy PAGE Gel Fast Preparation Kit (SEVEN BIOTECH, Beijing, China).

### 4.9. Transwell Assay

Migration: The cells that had been starving for 12 h were prepared for suspension. Then, 100 μL of suspension containing 1 × 10^5^ cells was inoculated in the upper chamber, and 500 μL of medium containing 20% FBS was added to the lower chamber. After 24 h of routine culture, the chamber was fixed with 4% PFA for 15 min. After that, the cells on the upper compartment were scraped off with a cotton wand. Finally, the lower compartment was stained with crystal purple for 10 min. The results were observed under an inverted microscope.

Invasion: The collagen was prepared into 0.5mg/mL working solution with basic medium, coated with the upper compartment surface of the chamber, and put into a 37 °C incubator for 30 min to solidify. Then, subsequent experiments were performed for migration.

### 4.10. Western Blotting

The total protein was extracted using the SevenFast total protein extraction kit from SEVEN BIOTECH (Beijing, China). The protein supernatant was determined using the BCA kit from Beyotime. The samples were incubated at 37 °C for 30 min, and the optical density (OD) was measured at 562nm to assess protein denaturation. Electrophoretic gels were prepared using the seven-color rapid gel dispensing kits. The proteins were then transferred onto PVDF membranes (Millipore, Merck KGaA, Darmstadt, Germany) after electrophoresis. The PVDF membranes were sealed with 5% skim milk at room temperature for one hour and incubated overnight at 4 °C with primary antibodies: *THBS1* (1:1000, #TD6848, Abmart, Shanghai, China); MMP2 (1:1000, #T57164, Abmart); MMP9 (1:8000, #ab76003, Cambridge, Abcam); MMP14 (1:2000, #T55236, Abmart); PD-L1 (1:1000, #TM033179, Abmart); PCNA (1:2000, #TA0239, Abmart); p-AKT (Ser473, 1:1000, #T40067, Abmart); AKT1/2/3 (1:1000, #T55561, Abmart); and GAPDH (1:10000, #ab8245, Abcam). On the second day, after recovering the primary antibody, the PVDF membranes were washed three times with TBST for 10 min each and then incubated with the secondary HRP conjugated antibody (oat anti-rabbit or goat anti-mouse, 1:8000; ZSGB BIO, Inc.) at room temperature for 1 h. Finally, the PVDF proteins were developed using the Supersignal West Femto Kit (cat. no. 34094; Thermo Fisher Scientific, Inc.) and scanned with an imager (ChemiDoc™ Touch Imaging System; Bio-Rad Laboratories, Inc.). Densitometric analysis was performed using Image J software (version 1.51j8; National Institutes of Health).

### 4.11. Wound Healing Assay

The transfected OSCC cells were seeded onto a six-well plate at a density of 5 × 10^5^ cells. Once the cells adhered stably to the plate, they were treated with the corresponding inhibitors based on their respective groups. A 200-μL pipette tip was used to create a vertical scratch across the bottom of the plate. The scratch was then rinsed three times with PBS to remove any remaining cells, after which the cells were cultured in a serum-free medium. Images were captured at 0 h and 12 h using a phase microscope from Olympus Corporation, Tokyo, Japan. Finally, Image J software was utilized for image analysis.

### 4.12. Cell Proliferation Assay

We evaluated OSCC cell proliferation using a BeyoClick™ EdU-594 assay kit (Beyotime, Shanghai, China). After cell transfection, the appropriate group was treated with inhibitors, and EdU was added to label tumor cells for 2 h. After the treatment, the cells were fixed with 4% PFA for 15 min and cleaned three times with PBS for 5 min each time. Then, the cells were treated with PBST for 15 min and washed three times with PBS for 5 min each time. The EdU reaction solution was incubated at 37 °C for 30 min according to the instructions. After washing with PBS 3 times, the nuclei were stained with a mounting medium containing DAPI for 5 min at room temperature, and cells were observed by a fluorescence microscope.

### 4.13. Cell Immunofluorescence Staining Assay

Cells were fixed with 4% PFA at room temperature for 15 min, followed by three 5 min washes with PBS. Subsequently, they were treated with PBST for 15 min and again washed three times with PBS for 5 min each. The cell slides were blocked with 5% normal goat serum (Abbkin, Wuhan, China) at room temperature for 1 h. The primary antibodies, including cortactin (1:200, #T55646, Abmart) and p-AKT (1:200, #T40067, Abmart), were incubated overnight at 4 °C. The next day, the primary antibodies were recovered, and then the cells were washed three times with PBS for 5 min each. Dylight 488 secondary antibodies (Abbkin) were incubated at 37 °C for 1 h, followed by three 5 min washes with PBS at the end. If necessary, F-actin staining was performed using rhodamine-conjugated phalloidin (1:400; Yeasen Biotechnology Co., Ltd., product no. 40734ES75) for 1 h at 37 °C. Finally, the cells were sealed using SuperKine™ Enhanced Antifade Mounting Medium with DAPI (Abbkin).

### 4.14. Statistical Analysis

All statistical analyses of the RNA-seq data were performed using R 4.2.2. Two subgroups were compared using Wilcoxon’s signed-rank test. Kaplan–Meier estimates, performed using the “survivor” and “survminer” R packages, were used to demonstrate the difference in survival between the high and low subgroups of ECM, as well as between the high-risk and low-risk subgroups. The independent predictive value of the clinical indicators was confirmed through univariate and multivariate Cox regression models. The “timeROC” R package was utilized for calculating the area under the curve, illustrating the receiver operating characteristic (ROC) curve, and assessing the effectiveness of the matrix risk score. The relationship between these two values was analyzed through the Pearson correlation test. All experiments were repeated at least 3 times, and the data are presented as the mean ± SD. A one-way analysis of variance, followed by Tukey’s post hoc test, was conducted for multiple comparison tests. If *p* < 0.05, the difference was considered statistically significant. The level of statistical significance was set at *p* < 0.05. The symbols, *, **, and *** represent *p* < 0.05, *p* < 0.01, and *p* < 0.001, respectively.

## Data Availability

The data that support the findings of this study are available from the corresponding author upon reasonable request.

## Supplementary Materials

The following supporting information can be downloaded at: https://www.mdpi.com/article/10.3390/ijms241713312/s1.
