# Supplementary material for: THBS1-Mediated Degradation of Collagen via the PI3K/AKT Pathway Facilitates the Metastasis and Poor Prognosis of OSCC
Source: Int J Mol Sci. 2023 Aug 28;24(17):13312. doi: 10.3390/ijms241713312 (PMC10488045; doi:10.3390/ijms241713312)
Supplement: Supplementary file 1 [file ijms-24-13312-s001.zip › ijms-2531076-supplementary.pdf]

THBS1-Mediated Degradation of Collagen via the PI3K/AKT Pathway Facilitates the Metastasis and Poor Prognosis of OSCC

Zhihao Wen [1,†], Yuxiao Zhang [1,2,3,†], Xiangyao Wang [1,2,3], Yaxin Wu [1,2,3], Jing Mao [1,2,3], Qilin Li [1,2,3,*] and Shiqiang Gong [1,2,3,*]

Affiliations

1 Department of Stomatology, Tongji Hospital, Tongji Medical College, Huazhong University of Science and Technology, Wuhan 430030, China

2 School of Stomatology, Tongji Medical College, Huazhong University of Science and Technology, Wuhan 430030, China

3 Hubei Province Key Laboratory of Oral and Maxillofacial Development and Regeneration, Wuhan 430022, China

* Correspondence: qilinli@tjh.tjmu.edu.cn (Q.L.); gsq@hust.edu.cn (S.G.); Tel.: +86-027-8366-3225 (S.G.)

† These authors contributed equally to this work.

Supplementary materials

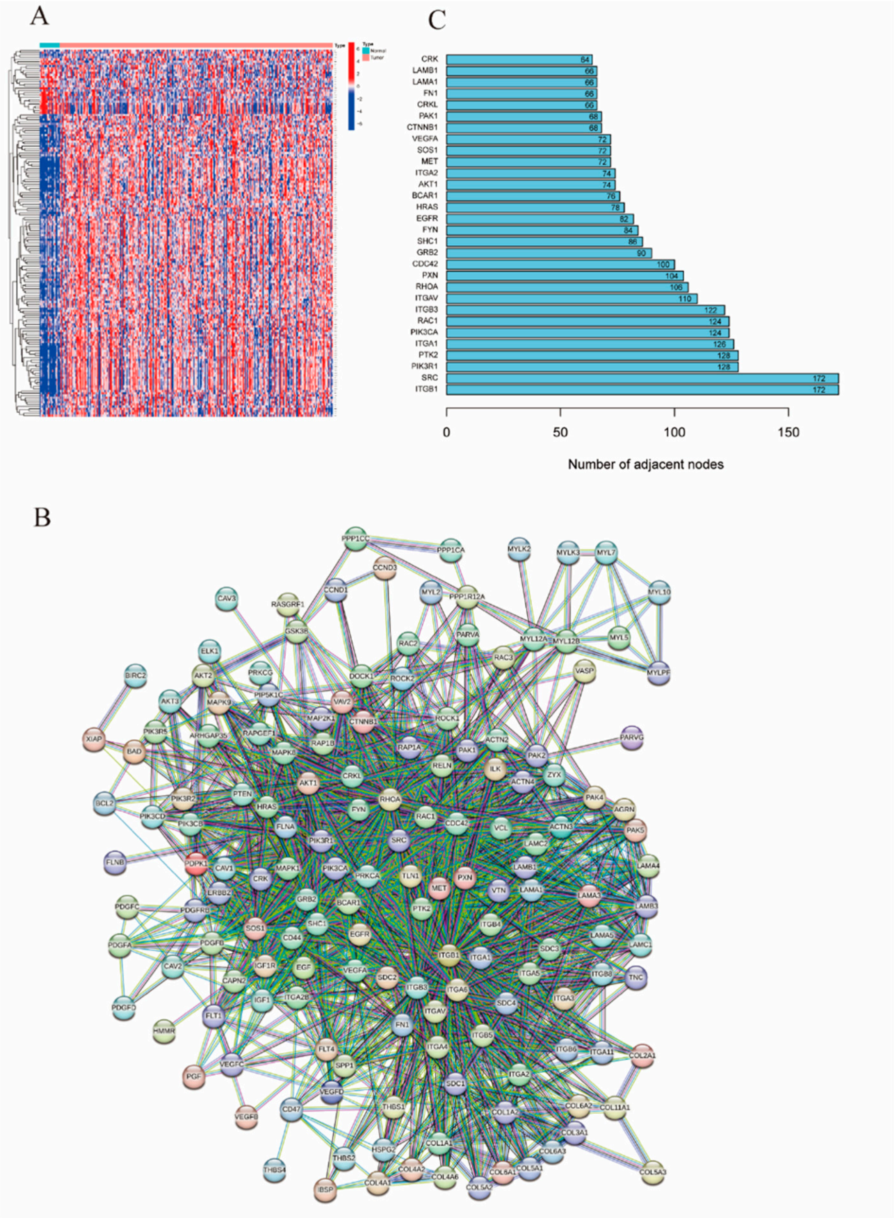

Figure S1. Identification of ECM-related DEGs in OSCC

(A) The heatmap of the expression of ECM-related genes in OSCC samples and para-cancerous normal samples. (B) The protein-protein interaction (PPI) analysis of ECM-related DEGs in OSCC patients. The cutoff for the PPIs network was set at the interaction nodes with scores > 0.95. (C) The top 30 key genes analyzed by PPI analysis of ECM-related DEGs in OSCC patients, including ITGB1, SRC, PIK3R1, PTK2, ITGA1, PIK3CA, RAC1, ITGB3, ITGAV, RHOA, PXN, CDC42, GRB2, SHC1, FYN, EGFR, HRAS, BCAR1, AKT1, ITGA2, MET, SOS1, VEGFA, CTNNB1, PAK1, CRKL, FN1, LAMA1, LAMB1, CRK.

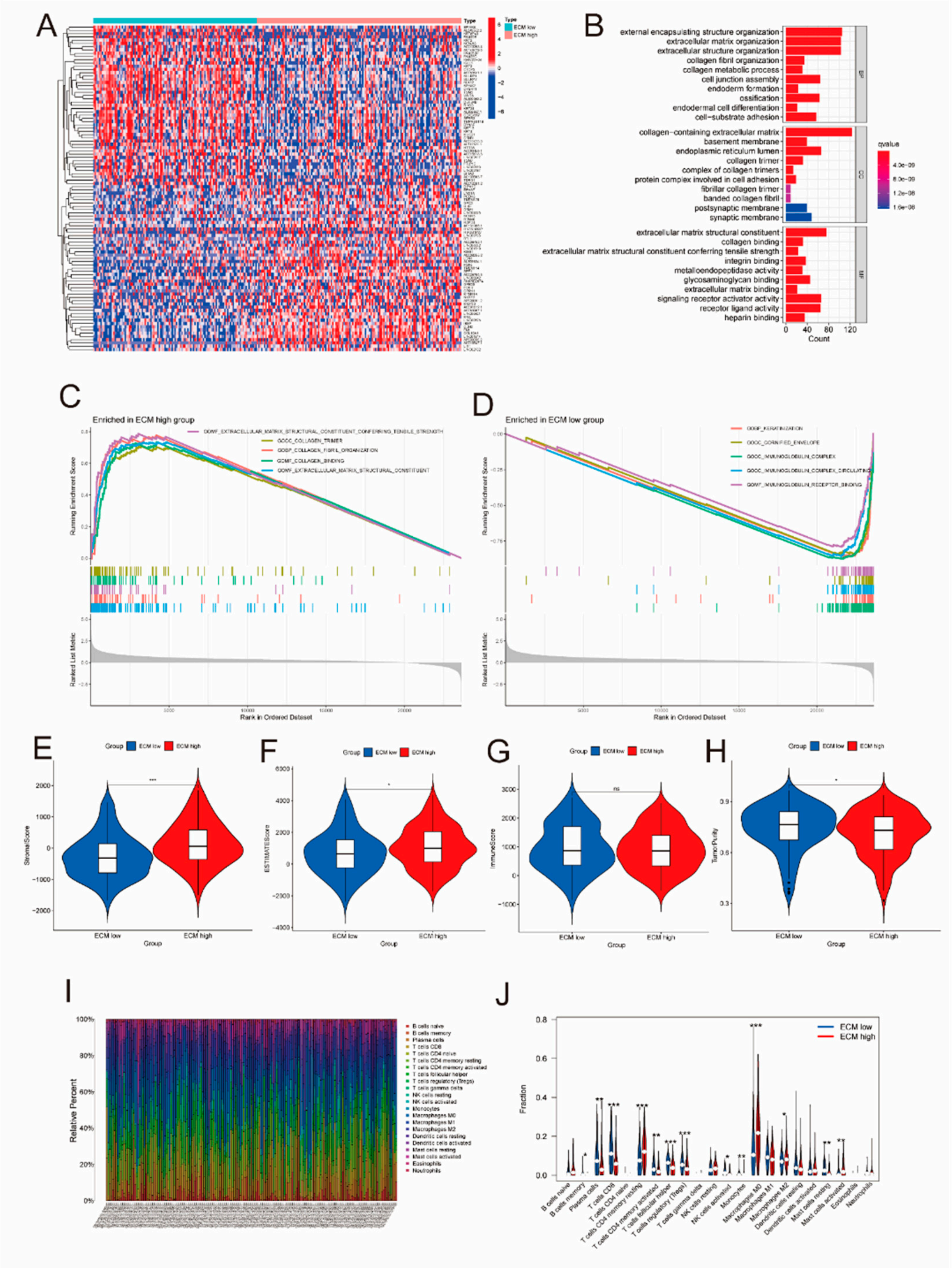

Figure S2. Characteristics of immune infiltration in different ECM clusters of OSCC

(A) Heatmap of the top 50 most significantly enriched DEGs in the ECM high and ECM low clusters. (B) Gene ontology (GO) enrichment analysis of DEGs in the ECM high and ECM low clusters. (C-D) Gene Set Enrichment Analysis (GSEA). The five most significant functions enriched in the ECM high (C) and ECM low (D) clusters in GSEA enrichment analysis. (E-H) Distribution of stromal score (E), Estimate score (F),

immune score(G), and tumor purity(H) in the ECM low and ECM high groups of the TCGA dataset. (I) Relative proportions of immune cell distribution in OSCC samples. (J) Differences in the proportion of immune cells in the ECM high and ECM low groups.

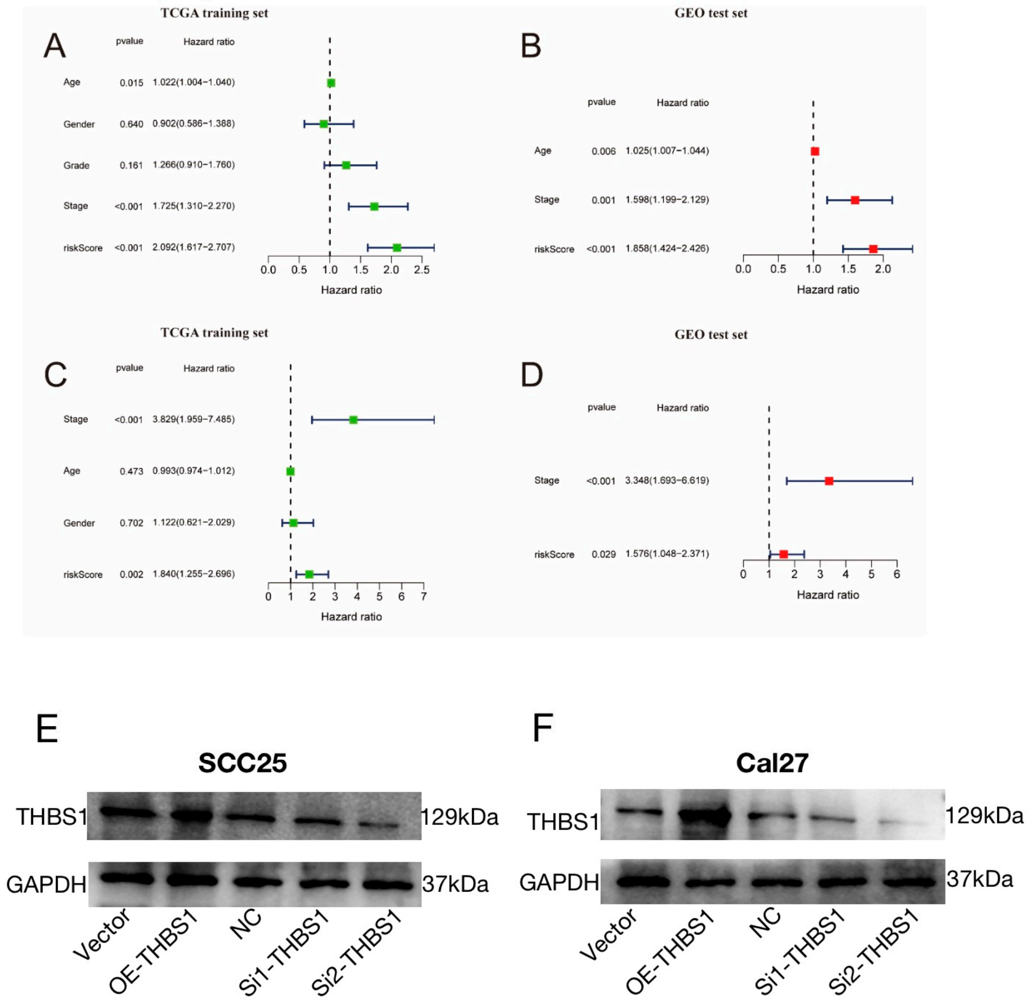

THBS1-siRNA1 (Si1-THBS1)	5′-GGAGTTCAGTACAGAAATA-3′
THBS1-siRNA 2 (Si2-THBS1)	5′-GTACAGAAATAACGAGGAA-3′

Figure S3. The ECM risk score is an independent prognostic factor for OSCC. (A) Univariate Cox regression and (B) multivariate Cox regression analysis of OSCC in the training cohort (TCGA dataset). (C) Univariate Cox regression and (D) multivariate Cox regression analysis of OSCC patients' overall survival in the test cohort (GEO dataset). (E, F) THBS1 overexpression and THBS1 expression efficiency after transient transfection of two SiTHBS1 were detected by WB.

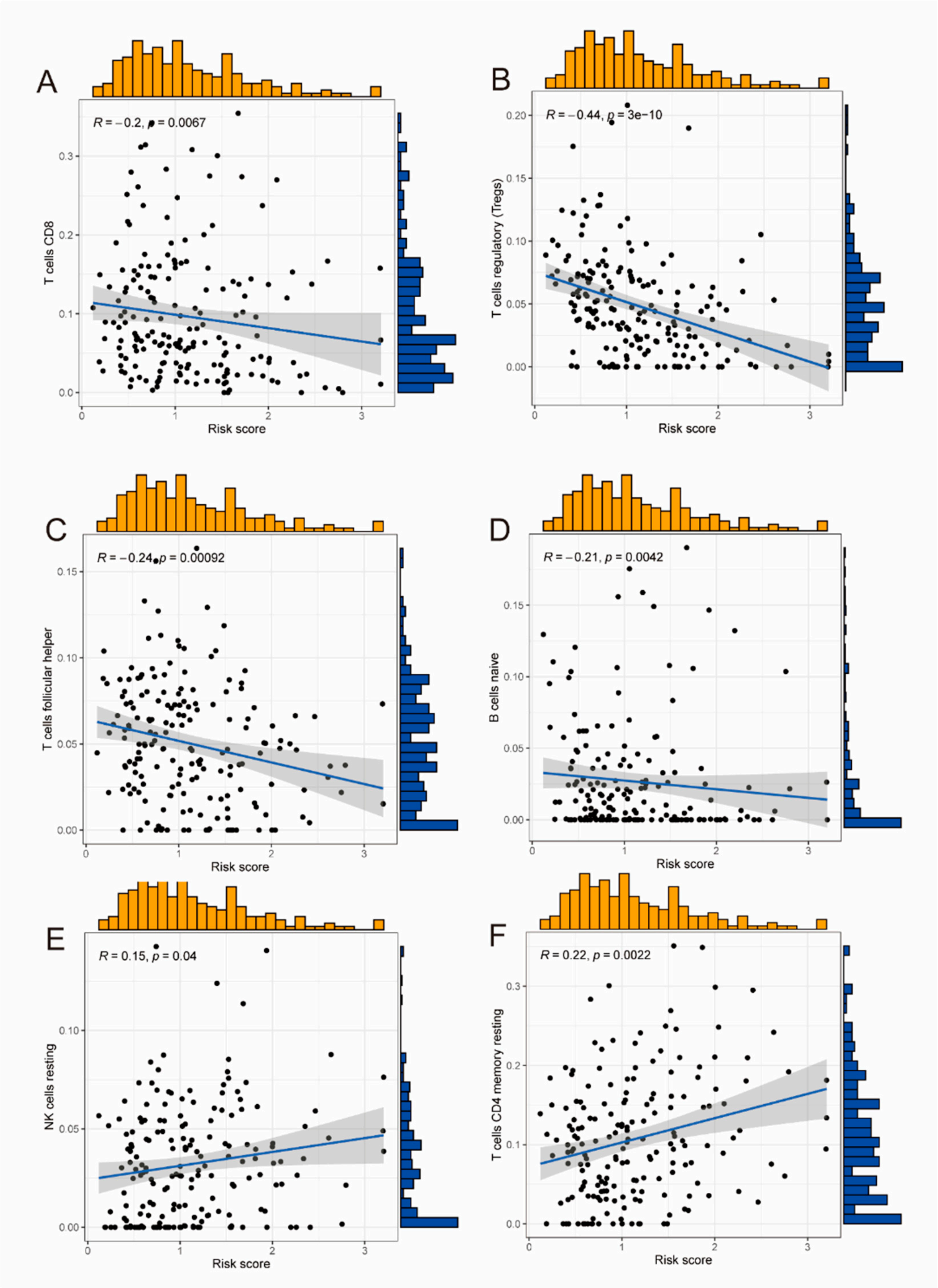

Figure S4. Correlation analysis of ECM risk scores and immune infiltration in OSCC

(A-F) Relationship between the ECM Risk score and immune cells of OSCC cases in the TCGA dataset: (A) CD8+T cells, (B) T cells regulatory (Tregs), (C) T cells follicular helper, (D) B cells naïve, (E) NK cells resting, (F) T cells CD4 memory resting.

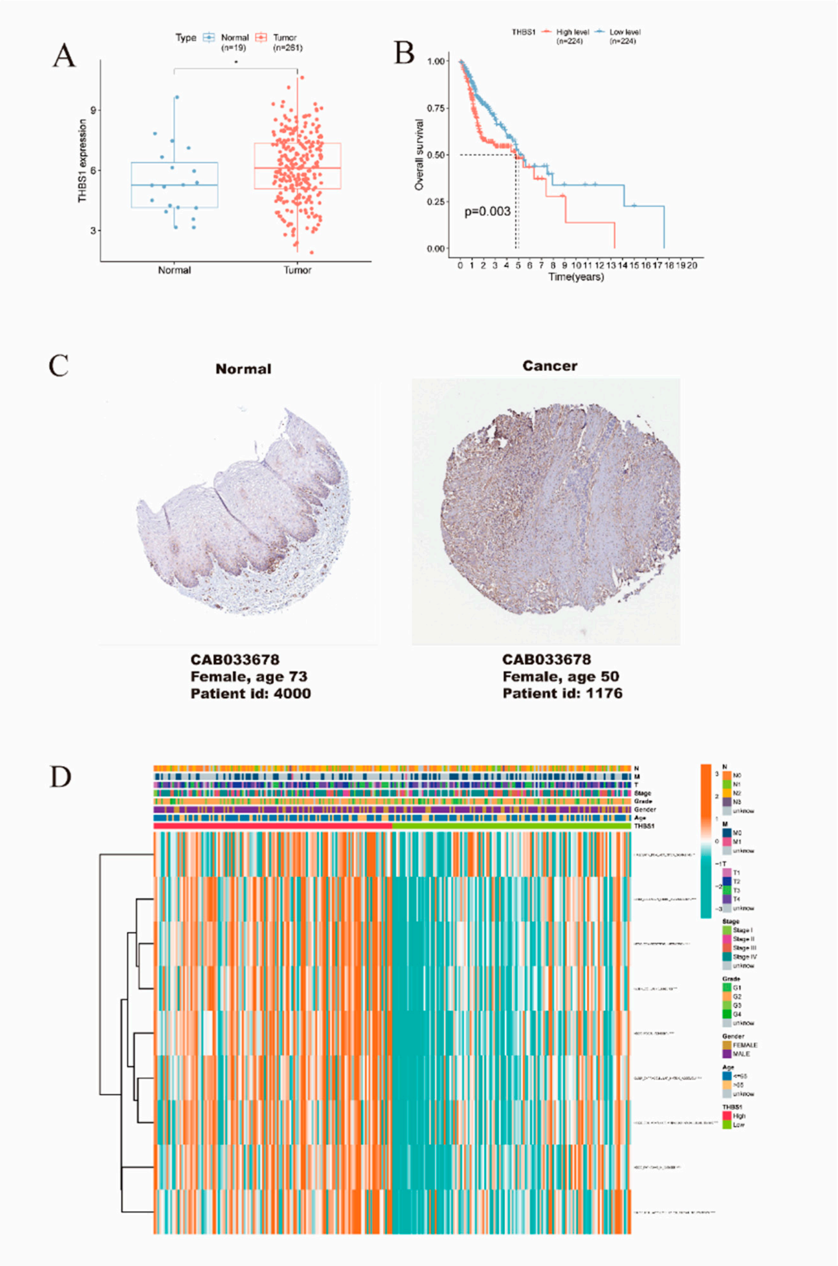

Figure S5. Ectopic expression of THBS1 is correlated with clinical parameters of OSCC

(A) THBS1 levels in OSCC samples grouped by cancer and para-cancerous status in TCGA. (B) Kaplan-Meier survival analysis of THBS1 high and THBS1 low groups. (C) Immunohistochemical maps from The Human Protein Atlas database (https://www.proteinatlas.org) for both normal and OSCC tumor tissues. (D) GSVA heatmap showing functional pathways significantly associated with THBS1 in TCGA (Hallmark PI3K pathway, GO ECM-related functions, and KEGG cancer-related pathways).
